# Reduced Population Control of an Insect Pest in Managed Willow Monocultures

**DOI:** 10.1371/journal.pone.0005487

**Published:** 2009-05-08

**Authors:** Peter Dalin, Oskar Kindvall, Christer Björkman

**Affiliations:** 1 Department of Ecology, Swedish University of Agricultural Sciences, Uppsala, Sweden; 2 Swedish Species Information Centre, Swedish University of Agricultural Sciences, Uppsala, Sweden; University of Lancaster, United Kingdom

## Abstract

**Background:**

There is a general belief that insect outbreak risk is higher in plant monocultures than in natural and more diverse habitats, although empirical studies investigating this relationship are lacking. In this study, using density data collected over seven years at 40 study sites, we compare the temporal population variability of the leaf beetle *Phratora vulgatissima* between willow plantations and natural willow habitats.

**Methodology/Principal Findings:**

The study was conducted in 1999–2005. The density of adult *P. vulgatissima* was estimated in the spring every year by a knock-down sampling technique. We used two measures of population variability, CV and PV, to compare temporal variations in leaf beetle density between plantation and natural habitat. Relationships between density and variability were also analyzed to discern potential underlying processes behind stability in the two systems. The results showed that the leaf beetle *P. vulgatissima* had a greater temporal population variability and outbreak risk in willow plantations than in natural willow habitats. We hypothesize that the greater population stability observed in the natural habitat was due to two separate processes operating at different levels of beetle density. First, stable low population equilibrium can be achieved by the relatively high density of generalist predators observed in natural stands. Second, stable equilibrium can also be imposed at higher beetle density due to competition, which occurs through depletion of resources (plant foliage) in the natural habitat. In willow plantations, competition is reduced mainly because plants grow close enough for beetle larvae to move to another plant when foliage is consumed.

**Conclusion/Significance:**

To our knowledge, this is the first empirical study confirming that insect pest outbreak risk is higher in monocultures. The study suggests that comparative studies of insect population dynamics in different habitats may improve our ability to predict insect pest outbreaks and could facilitate the development of sustainable pest control in managed systems.

## Introduction

The push to reduce CO_2_ emissions from fossil fuels in favor of bioenergy together with the Earth's growing human population leads to enhanced land transformations and intensifications of forest and agricultural systems [1]–[2]. Agricultural crops and forest trees are increasingly being planted in large monocultures. The transformation of natural habitats into monocultures may increase plant productivity, but may at the same time result in losses of important ecosystem services, such as the control of insect pest populations [3]–[4]. The vulnerability of intensively managed plant systems to insect pest outbreaks may therefore increase in the future, which could also be further enhanced by the ongoing global warming [5]. One overriding hypothesis to why plant monocultures should be more susceptible to insect outbreaks is that the factors regulating insect populations in natural habitats are often altered when the habitat is transformed, which can result in larger population fluctuations and ultimately insect outbreaks [6]. Empirical studies testing this hypothesis are however rare, as well as knowledge about the mechanisms bringing about population stability in natural systems.

For insect pests causing damage to forest trees and crop plants when reaching high densities, monocultures may provide improved conditions for population growth. Monocultures consisting of plants of high and even quality for insects have potential to harbor large insect populations. Moreover, with few other plant species interfering with insect host plant selection behaviors, monocultures can facilitate for insects finding their host plants and enhance their dispersal from plant to plant, which could reduce competition for resources [7]. Transforming natural habitats into monocultures also leads to reduced biodiversity [8], and changes in food web interactions [9]. Important interactions with natural enemies may therefore change, leading to altered survival and population growth of herbivorous insects, mediated by reductions in both the diversity and the abundance of predators and parasitoids [3],[10]. The sum of such changes would be increased risk of insect outbreaks.

Since the early 1990's, willows (mainly *Salix viminalis*) have been planted in monocultures on agricultural land in Sweden and other parts of Europe for biomass production in an attempt to reduce the dependence upon fossil fuels and to reduce atmospheric CO_2_. However, defoliation by willow leaf beetles (Coleoptera: Chrysomelidae) during population outbreaks can cause substantial plant growth reductions [11]. The leaf beetle *P. vulgatissima* is also a common herbivore on the willow *S. cinerea* growing naturally in northern Europe. The two willow species share many features and are chemically very similar [12], as indicated by the fact that larval performance of *P. vulgatissima* do not differ significantly between *S. cinerea* and *S. viminalis* [13]. Comparing population dynamics of willow leaf beetles between plantations and natural willow stands, thus, provides an opportunity to test whether insect outbreak risk is higher in managed systems, because (i) willow plantations are even-aged stands usually consisting of only one willow clone or cultivar, whereas natural willow stands can be more diverse in terms of plant genotypes, species, age and structure, (ii) high population densities of *P. vulgatissima* can be found on both willow species [14]–[15].

The purpose of the current study was to compare the temporal variability in the density of the leaf beetle *P. vulgatissima* between 20 willow plantations and 20 natural willow stands over a seven year period (1999–2005). We analyzed the relationship between population density and variability to discern potential underlying processes influencing stability in the two habitats. We also estimated the density of important predators to investigate if variation in leaf beetle density among willow stands could be explained by natural enemy impact.

## Materials and Methods

In 1999, we initiated a study to compare the temporal population variability in the density of the leaf beetle *Phratora vulgatissima* over seven years (1999–2005) between 20 willow biomass plantations (*Salix viminalis*) and 20 natural willow stands (*Salix cinerea*) in Central Sweden. All willow stands included in the study were located within a 40 km radius around the city of Uppsala (59°51′N, 17°38′E), and should therefore have been exposed to similar climate conditions. Distance between individual stands was at least 1 km. The willow stands were selected without any knowledge about beetle density in previous years. Willow plantations consisted of *Salix viminalis*, except for one plantation with a few rows of *S. dasyclados*. No herbicides or pesticides are used in willow plantations. The natural *S. cinerea* stands were on average smaller (mean: 0.3 ha, range: 0.1–0.8 ha) than plantations (mean: 4.8 ha, range: 0.1–15.0 ha). Stand area did, however, not seem to affect leaf beetle density or population variability in the two habitats (see below).

The leaf beetle *Phratora vulgatissima* has one generation per year (univoltine) in Sweden. Adult density was estimated in late May or beginning of June after adult emergence from overwintering. Methods for estimating beetle density have been described before [14]–[15]. In principle, density is estimated by a knock-down technique, using a cylinder or plastic bucket to knock off all insects from 35 cm sections of plants containing current year shoots. The purpose is to take many samples within each willow stand to reduce possible sampling error due to spatial variation in beetle density within the stands. The average number of samples taken in each stand and year was 65 (range: 30–125). A minimum of 30 samples was selected based upon previous studies showing that 20–25 samples are needed to receive a stable estimate of beetle density [14]. We also estimated density for three common predators: *Orthotylus marginalis* Reut. (Heteroptera: Miridae), *Closterotomus fulvomaculatus* De Geer (Heteroptera: Miridae) and *Anthocoris nemorum* L. (Heteroptera: Anthocoridae) [16]. Other types of predatory arthropods, such as ladybugs and spiders, may also feed on leaf beetles but were relatively uncommon in the study. For each sample, the number of beetles and predators were counted and released back to the base of the plants. Density was calculated as the average number of individuals per 35 cm sections of willow shoots.

Two different measures of population variability were used. Coefficient of Variation (CV), which is the most common and widely accepted method for measuring population variability [17], was calculated for each willow stand by dividing the standard deviation of density with the mean density of *P. vulgatissima* over the seven year period. Zero counts (i.e. no beetles found; five zero counts in total) were transformed to the lowest detectable density by dividing the number 1 with the number of samples taken in the focal willow stand. We thereby assumed that *P. vulgatissima* was present in each stand every year but that they sometimes occurred at densities too low to be detected by our sampling method. Population variability (PV), which is a relatively new method proposed for measuring temporal variation [18], quantifies variability as the average percentage difference between all combinations of data points and was calculated using MATLAB. PV is supposed to be less sensitive to extreme events, such as zero counts and large deviations from the mean, than CV. This method is therefore especially appropriate when comparing variability of populations experiencing different types of dynamics because PV measures variability on a proportional scale. We used the same data points for calculating PV and CV and, thus, used transformed zero counts in the analyses.

### Statistical analyses

Statistical tests were performed using SAS® (Version 9.1 for Windows, SAS Institute, Inc., Cary NC, USA). We first compared CV and PV between willow plantations and natural stands using t-tests (PROC MEANS in SAS). We then fitted regression models to investigate relationships between log_10_ mean beetle density and population variability in the two habitats (PROC REG in SAS). Polynomial regressions were then done to compare linear to non-linear relationships using a step-up approach to find the best fitting model, starting with linear (x^1^) and ending with x^8^ polynomial. For both CV and PV, we found that only relatively simple, and seemingly appropriate, models were significant (see results section). Mean densities of leaf beetles and predators were compared between habitats using t-tests (PROC MEANS in SAS). Because the three heteropterans attack the same life-stages of leaf beetles (eggs and larvae) and have similar consumption rates [16], the densities of predators were pooled together in the analyses. Pearson's correlation analyses were used to study relationships between predator and beetle density in the two habitats. The effects of stand area on densities of beetles and natural enemies, and the effect of stand area on beetle variability, were also analyzed using Pearson's correlations (PROC CORR in SAS).

## Results

**Figure 1:**
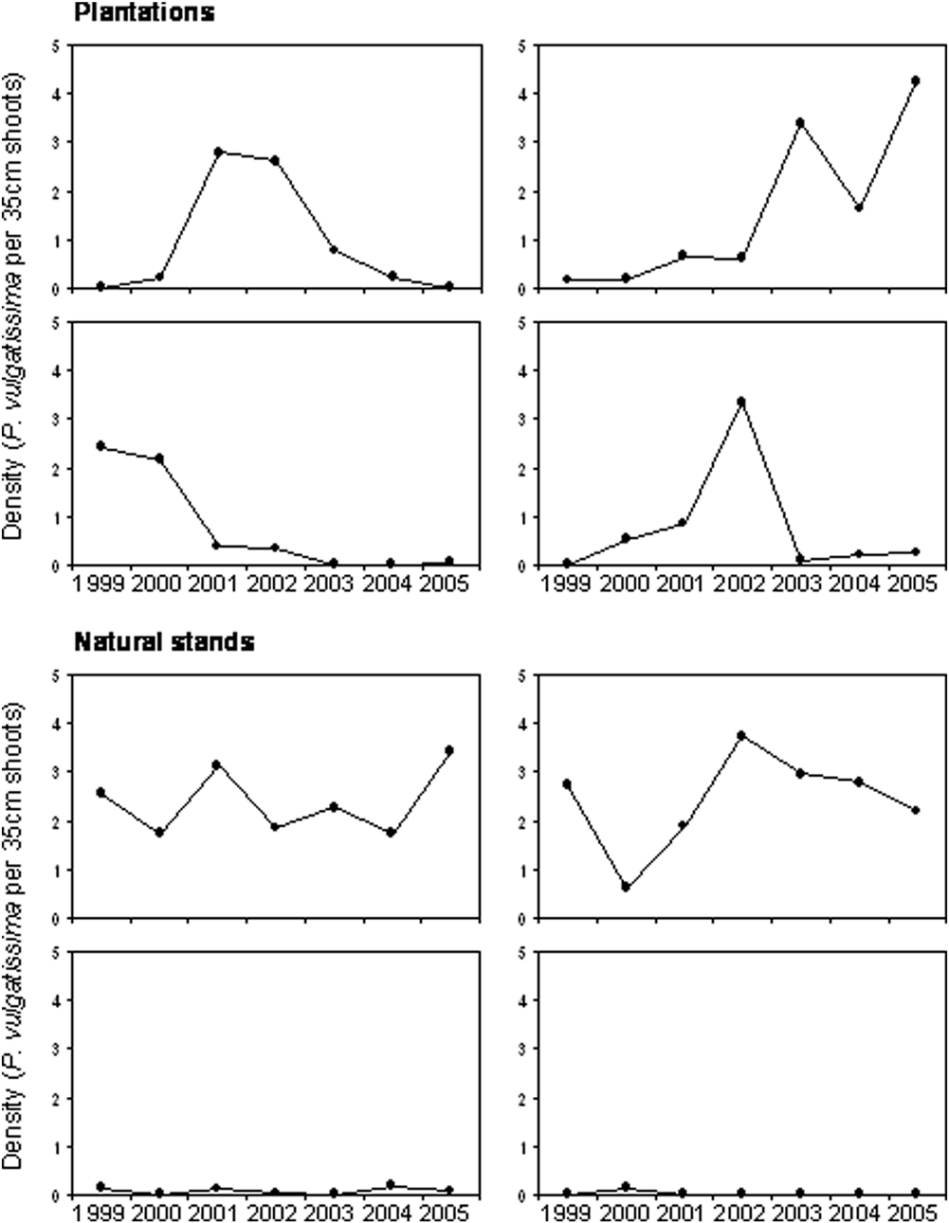
Population dynamics of *Phratora vulgatissima* in plantations (upper four) and natural stands (lower four). The four examples from willow plantations represent outbreak dynamics (fluctuations between low and high densities), whereas the examples from natural stands represent fluctuations around relatively stable high (upper two) and low (bottom two) mean densities. Density is measured as the average number of adult beetles per 35 cm willow shoots in the spring.

Density of the leaf beetle *Phratora vulgatissima* showed greater temporal variability in willow plantations than in natural willow stands during the study period (1999–2005). Means±standard errors (S.E.) are presented throughout the results section. Coefficient of variation (CV) was 106±11 in plantations, and 76±10 in natural stands (*t* = 2.05, *P* = 0.047, *d.f.* = 38). Population variability (PV) was 60±4 in plantations, and 42±3 in natural stands (*t* = 3.53, *P* = 0.001, *d.f.* = 38). The average density of leaf beetles during the study period did, however, not differ between plantations and natural stands (*t* = 0.01, *P* = 0.925, *d.f.* = 38). Average density of adult *P. vulgatissima* per 35 cm sections of plants in the spring was 0.347±0.092 in plantations, and 0.365±0.164 in natural stands. An estimated density of >1–2 adult beetles in the spring normally means the willow stand will become heavily defoliated later in the season when the larval generation feeds on the plants [15]. Such high densities were found in both natural and managed willow stands (Fig. 1).

**Figure 2:**
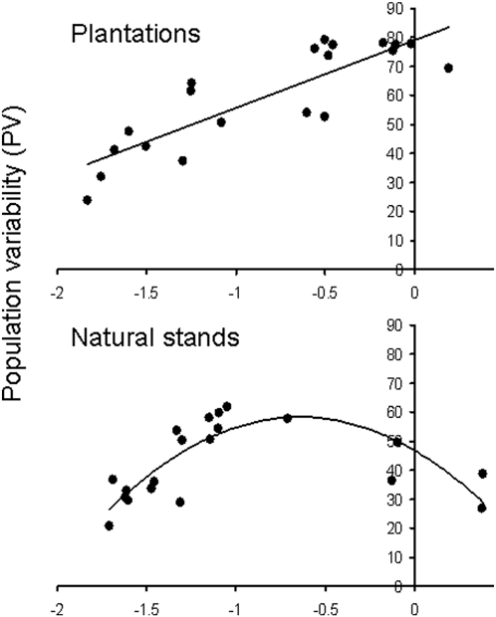
Regression models between population variability and mean density of *P. vulgatissima*. Models are fitted to the relationships between population variability (PV) and mean density in willow plantations (upper figure) and natural willow stands (lower figure).

The relationship between PV and density of *P. vulgatissima* was positively linear in plantations (Fig. 2, top graph; PV = 23.38×log_10_ [beetle density]+78.93; F_1, 19_ = 48.34, *r^2^* = 0.73, *P*<0.001), whereas the relationship was significantly curvilinear in the natural habitat (Fig. 2, bottom graph; PV = −27.92×log_10_ [beetle density]^2^−35.81 log_10_ [beetle density]+46.90, F_2,19_ = 17.04, *r^2^* = 0.67, *P*<0.001). Similar relationships were found for CV; linear in willow plantations (CV = 48.26×log_10_ [beetle density]+145.84; F_1, 19_ = 11.25, *r^2^* = *P* = 0.004), and curvilinear in natural stands (CV = −9.28×log_10_ [beetle density]^2^−103.56×log_10_ [beetle density]+71.64; F_2, 19_ = 5.25; *r^2^* = 0.38; *P* = 0.016). There were no significant relationships between beetle density and stand area (*r* = −0.23, *P* = 0.334, *n* = 20), or between population variability and stand area (PV: *r* = 0.29, *P* = 0.216, *n* = 20; CV: *r* = 0.28. *P* = 0.241, *n* = 20) in willow plantations. Similar non-significant relationships were found in the natural habitat.

**Figure 3:**
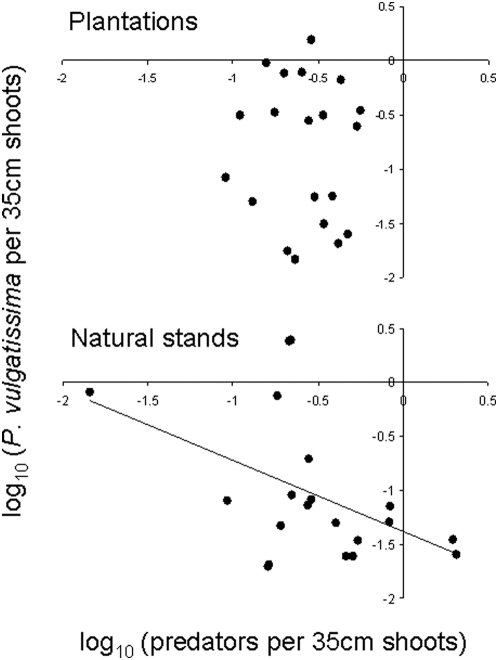
Correlations between *P. vulgatissima* and natural enemies. Each data point represent the mean density of the three Heteropteran predators *Orthotylus marginalis*, *Closterotomus fulvomaculatus* and *Anthocoris nemorum* added together, and the leaf beetle *Phratora vulgatissima* over seven years (1999–2005) in willow plantations (top figure) and natural willow stands (bottom figure). There was a significant negative correlation in the natural habitat (*r* = −0.46, *P* = 0.019, *n* = 20), but not in plantations (*r* = –0.06, *P* = 0.40, *n* = 20).

The density of the most common predators (*i.e*. the three Heteropterans *Orthotylus marginalis*, *Closterotomus fulvomaculatus* and *Anthocoris nemorum* [16], added together), did not show any significant difference between willow plantations and natural willow stands (*t* = 1.55, *P* = 0.130, *d.f.* = 38). Predator density was, however, more variable among natural stands than among willow plantations (Fig. 3). The predators were sometimes more abundant than leaf beetles, especially in some of the natural stands (average densities: 0.296±0.031 in plantations, and 0.498±0.127 in natural stands). We found a negative correlation between leaf beetles and predators in the natural habitat (Fig. 3, bottom graph; *r* = −0.46, *P* = 0.019, *n* = 20), but not in willow plantations (Fig. 3, top graph; *r* = −0.06, *P* = 0.40, *n* = 20). Stand size seemed to have a positive effect on predator density in willow plantations (*r* = 0.61, *P* = 0.004, *n* = 20) but not in natural habitats (*r* = 0.23, *P* = 0.324, *n* = 20).

## Discussion

We found that populations of the leaf beetle *Phratora vulgatissima* showed greater temporal variation in willow plantations than in natural willow stands, although the average density of beetles was similar in the two habitats during the study period. High densities were observed in both habitats, resulting in plants being heavily defoliated by the beetles. In willow plantations, the beetles fluctuated widely between low (<0.2 beetles/shoot) and high (>2 beetles/shoot) densities in five of the twenty plantations studied (see examples in Fig. 1, top four graphs). This type of outbreak dynamics was not observed in any of the natural stands. In the natural habitat, the beetles were either fluctuating around relatively stable high or stable low densities throughout the whole study period (see examples in Fig. 1, bottom four graphs). These results suggest that, although leaf beetles may defoliate plants both in willow plantations and natural habitats, the populations were more strongly regulated within upper and lower density limits in the natural habitat and, thus, the risk of extreme population fluctuations (e.g. outbreak risk) was higher in willow plantations.

We will propose two main explanations to why leaf beetle populations fluctuated more in willow plantations. These explanations should mainly be treated as hypotheses that need further testing in the field. The relatively stable population dynamics observed in the natural habitat can be achieved by density dependent population control [19], through effects from (i) natural enemies (functional and/or numerical responses [20]), (ii) plant quality (induced resistance [21]) and (iii) competition [22]. We argue that all three processes may have influenced stability in the natural habitat, although natural enemies and competition seem to be the main processes causing stability in the system. We also suggest that the effects of natural enemies and competition were operating at different levels of beetle density.

The most common natural enemies attacking *P. vulgatissima* have been identified to be three species of predatory bugs (Heteroptera); two mirids and one anthocorid, which attack eggs and young larvae of leaf beetles [16]. Survival and population growth of *P. vulgatissima* is oftentimes negatively correlated with the density of predatory bugs [14], [23], revealing that predation is an important mortality factor affecting the population dynamics of *P. vulgatissima* [15]. Although the average predator density did not differ between plantations and natural habitats, we found greater variation in predator density among the natural stands with very high densities in some of the natural stands. There was also a negative correlation between leaf beetles and predators in the natural habitat, which was not observed in willow plantations. This suggests that natural enemies explained some of the variation in beetle density observed among the natural stands. The leaf beetle fluctuated around stable low densities in those natural stands where the density of predatory bugs was high, implying that predators can control leaf beetle populations at low densities when predators are abundant.

The reduced ability of predatory bugs to control leaf beetles in plantations can be explained by the fact that willow plantations are harvested every 4–5 years, which has been shown to reduce the density of predatory bugs [14]. All willow plantations were harvested at least once during the study period and takes place in the wintertime when the two mirid species overwinter as eggs on the plants. These predators are therefore removed when the plantation is harvested. The harvesting regime is, however, not expected to have any direct effects on leaf beetle populations because *P. vulgatissima* leave the willows in the autumn for overwintering outside of plantations [24]. The beetles re-colonize the plantations again in the spring when the plants have re-sprouted after harvest and, thus, when foliage is available. The beetles will therefore be exposed to relatively low predation pressure the first year following harvest, although the density of predators and predation rates may increase in subsequent years [14]. It has been shown that pest control imposed by natural enemies is more common in natural habitats than in cultivated habitats [25], although the underlying mechanisms for stronger top-down control in natural habitats are oftentimes unknown. Our study shows that generalist predators sometimes occur at high densities on natural willows, which seem to prevent population increase of leaf beetles. Predation from predatory bugs also affects leaf beetles in plantations, but the intermediate disturbance regime from harvesting imply that leaf beetles are likely to escape the control [14]. The positive relationship between stand area and predator density in willow plantations suggest, however, that increased plantation size may have a positive effect on the predator populations.

The curvilinear relationship between CV and density in Fig. 2 shows that beetle populations became stable at high and low densities in the natural habitat. The most likely explanations for the stable dynamics at high density are: (i) competition for resources [22] and (ii) induced plant resistance [21]. Natural enemies occurred at too low densities in those natural stands with high beetle abundance to have any major impact on population stability (Fig. 3). Competition for resources seems to be the most likely reason because: (i) *P. vulgatissima* adults often aggregates on plants within willow stands in the spring and are attracted to volatiles emitted from damaged plants [26], and (ii) plants occur more patchily in the natural habitat with a larger plant-to-plant distance than in plantations. Thus, adult beetles will lay most of their eggs on a few plant individuals within the natural habitat. In addition, the relatively large distance between plants in the natural habitat mean that larvae cannot move between plants when foliage is consumed, resulting in increased competition (depletion of resources) on individual plants. In plantations, where the plants grow more closely together, both adults and larvae can move more easily between plants and are therefore less likely to become concentrated on certain plant individuals, which should reduce competition. In the natural stands, the populations were fluctuating at levels below the carrying capacity of the habitat because some plants are less utilized by the beetles. This type of self-regulation through variation in defoliation and competition within habitats is expected to facilitate temporal stability of insect populations [27]–[28], and our data seem to support this notion.

Population stability can also be influenced by induced plant defense responses [21], which have been documented in *S. cinerea* when attacked by *P. vulgatissima* [29], but are lacking in *S. viminalis* [30]. However, because of the relatively small effects of the induced plant responses on leaf beetle performance [29], it is difficult to foresee what effect it has on population stability in this system. This is also the only obvious difference between the two willow species that we have been able to document that may affect the performance of *P. vulgatissima*, especially at high densities. Thus, the fact that natural willow stands consisted solely of *S. cinerea* and the managed ones of *S. viminalis* may be less of a problem than intuitively thought. The similar average beetle densities reported here, and the similar performance reported previously [13] on the two willow species support this conclusion.

The mechanisms suggested here to affect population stability and outbreak risks of willow leaf beetles should be wise to take into account in forest management practices and conservation. The results from our habitat comparison suggest that generalist predators can control leaf beetle populations at low densities when predators are abundant. Conservation and management practices that enhance population growth of natural enemies inside plantations, as well as the movement of enemies into plantations from surrounding landscapes, could therefore facilitate sustainable pest control. The planting of trees in dense monocultures also increases the risk of large insect outbreaks because dense stands facilitate insect movement from tree to tree and, thereby, reduces competition and promotes population growth. Increased habitat heterogeneity and plant diversity may therefore reduce the risk of extremely high insect densities and could prevent widespread defoliation. Our findings suggest that comparative studies of insect population dynamics in different habitats will improve our ability to predict and prevent insect pest outbreaks in intensively managed forest and cropping systems as well as facilitating the development of sustainable insect pest management methods.
